# Supplementary material for: The braid index of DNA double crossover polyhedral links
Source: PLoS One. 2020 Feb 12;15(2):e0228855. doi: 10.1371/journal.pone.0228855 (PMC7015418; doi:10.1371/journal.pone.0228855)
Supplement: Attachment — Submitted filename: Revision_Report.pdf [file pone.0228855.s001.pdf]

Revision Report of
"The braid index of DNA double crossover polyhedral links"
by Xiaosheng Cheng and Yuanan Diao

We would like to thank the referees for the careful reading of our manuscript and many thoughtful comments. We hope we succeeded addressing all issues to the referees' satisfaction. The first referee asked us to go through our proofs carefully one more time, and we did. We are confident that our proofs are sound. In this report we outline what we have done to address the comments and suggestions provided by the second referee.

Part 1. Comments/suggestions by the second referee are:

General suggestions:

- It would be great if the authors could provide the description and comparisons of classes of knots that are at play, e.g. how large is the class of pseudo polyhedral knots compared to the double crossover ones? What are other topological properties of these knots that are known or of interest for applications?

- Can Section 3 have a more specific title or would the authors consider adding something to the title that would say that the results apply to a larger class of knots and links?

- Titles of Sections 3 and 4 seem indistinguishable. Is it fair to specify that Section 4 contains the proof?

- Suggestion: include notation for the link on Figure 3 and use it as a running example as it appears several times in the text, including page 9 (where in the paragraph starting with "For example, for the double.." it is not at all clear which graph G is used so it would be good to be able to refer to it).

Specific comments:

- Pg 2. Replace "terminologies" with terminology

- Pg 3. and Pg 5. Replace "It is well knowns" with something stating that according to Alexander's theorem

- Pg 3. Up to the authors but we suggest using HOMFLY-PT instead of HOMFLY to acknowledge all of the independent constructions

- Pg. 7 Fig. 7 Please add color or labels describing the correpsondence between Seifert circles and the vertices of the graph, crossings and edges... also add notation from the section 2.3 to the caption of Figure 7.

-Pg 7. "We know" can be safely removed from the first sentence of Ch 3.

-Pg 8. It is impossible to distinguish different Seifert circles in Figure 9. Color or some other tool should be used to make this clear.

-Pg 8 Caption of figure 9: please specify which link is in Figure 3 and change "in the top" to "on the top"

-Pg 9. "For example, for the double.." see general suggestions but definitely include figures.

-Pg 9. "Similarly " comes after examples- please specify

-Pg 9. "It is fairly easy" is not very useful as this usually implies a significant level of technicalities that the authors did not want to go into. Please modify or include a more specific description of what the generalized result would look like/depend on.

-Pg 10. In addition to changing the title please add a little description of this section in the beginning.

-Pg 11. Increase the size/thickness on Fig. 11, 13, and 14.

- pg 13: replace "the following lemma is well known .." with "Since the following well-known lemma is needed in the proof of the main Theorem 1 we state it here for completeness" or with a similar sentence.

- Pg 14. Theorem 2 provides a powerful tool for ? please make it a more complete sentence if possible. I am not sure how to parse "on the one hand" and "on the other hand" as these two statements read as "Theorem 2 is very powerful (at least partially) because the class of links it covers is large" and then it also has the main Theorem 1 as a simple corollary.

-Pg. 14. Can you provide more examples of knots and links in this class? Is there a database?

-Pg 15. stylistic suggestion: replace the first sentence of Section 5 with "In this paper we provide explicit formula" to avoid repeating the same phrase.

- Pg 16. Maybe replace: Thus we obtain ... with "the insights from the proof of Theorem 3...."

- Pg 16. Define all notation used in Theorem 4.

Part 2. This part addresses the general suggestions by the second referee.

1. This is a good question, but also a question that is difficult to address. In order to give a precise comparison between the two sets (the class of pseudo polyhedral knots and the class of the double crossover ones), one would need to do a precise count on the numbers of links in each class (or very good estimates of these numbers), presumably as functions of the minimum crossing numbers. However that is well beyond the scope of this paper. Thus we only provided the following paragraph at the end of the paper to address this:

We end this paper with the following remark. As we mentioned earlier, the class of pseudo polyhedral links is large compared to the double crossover ones. For example, for each template graph G that is k-regular, there is exactly one double crossover polyhedral link $L(G)$ with 4.5 turn using G as the template graph (with $c(L(G)) = 4kn(G) + 10e(G) = 18e(G)$), but the number of pseudo Type A double crossover polyhedral links with G as their template graph and with $18e(G)$ as their crossing number can be roughly estimated in the order of $3^{3e(G)}$. A precise enumeration is beyond the scope of this paper and will be addressed in a future work. This means that in general, the number of pseudo double crossover polyhedral links grows exponentially as a function of the crossing number. However, we need to point out that the class of pseudo polyhedral links is nonetheless a very special class of links among all links. It is a subset of the intersection of several well known classes of links: the alternating links, the special links (defined and studied by Murasugi in [30]) and the positive (negative) links. We note that although there have been studies concerning the braid index of links in these classes, these studies do not contain results that can be readily applied to the pseudo double crossover polyhedral links.

2 and 3. We added the following paragraph at the end of the introduction section to make it clear that Section 4 contains the more general results and the proofs:

The rest of the paper shall be organized in the following way. In the next section, we provide some necessary background knowledge, concepts and terminology in knot theory and graph theory. In Section 3, we outline the results for the double crossover polyhedral links. The reason for us to do so, instead of stating the theorems in the general cases only, is so that our reader familiar and interested in the applications of these types of links can easily comprehend our results and compare them with the previously known results. In Section 4, we state and prove our theorems under in the general cases. In the last section, we end the paper by showing how our approach and results in this paper may be used to provide alternative proofs for some previously known results in the case of double crossover polyhedral links with 4 turn, as well as obtaining some parallel (new) results for the case of double crossover polyhedral links with 4.5 turn.

We also changed the title of Section 4 to “The main results and proofs for the general cases”.

4. This is done for both links in Figure 2 and Figure 3. And the places where these links are used have been updated accordingly.

Part 3. This part addresses the specific comments by the second referee.

- Pg 2. Replace "terminologies" with terminology: Done.

- Pg 3. and Pg 5. Replace "It is well knowns" with something stating that according to Alexander's theorem: Done.

- Pg 3. Up to the authors but we suggest using HOMFLY-PT instead of HOMFLY to acknowledge all of the independent constructions: Changed.

- Pg. 7 Fig. 7 Please add color or labels describing the correpsondence between Seifert circles and the vertices of the graph, crossings and edges... also add notation from the section 2.3 to the caption of Figure 7. Changed.

-Pg 7. "We know" can be safely removed from the first sentence of Ch 3. Removed.

-Pg 8. It is impossible to distinguish different Seifert circles in Figure 9. Color or some other tool should be used to make this clear. Part of the graph is enlarged to show the details with color.

-Pg 8 Caption of figure 9: please specify which link is in Figure 3 and change "in the top" to "on the top": Specified and changed.

-Pg 9. "For example, for the double.." see general suggestions but definitely include figures. Changed.

-Pg 9. "Similarly " comes after examples- please specify: Changed.

-Pg 9. "It is fairly easy" is not very useful as this usually implies a significant level of technicalities that the authors did not want to go into. Please modify or include a more specific description of what the generalized result would look like/depend on. We deleted Corollary 2 and stated Corollary 1 in the general case for a k-regular graph, and provided the complete proof of it.

-Pg 10. In addition to changing the title please add a little description of this section in the beginning. This has been addressed by the response in 2-3 of Part 2 above.

-Pg 11. Increase the size/thickness on Fig. 11, 13, and 14. Done.

- pg 13: replace "the following lemma is well known .." with "Since the following well-known lemma is needed in the proof of the main Theorem 1 we state it here for completeness" or with a similar sentence. Changed.

- Pg 14. Theorem 2 provides a powerful tool for ? please make it a more complete sentence if possible. I am not sure how to parse "on the one hand" and "on the other hand" as these two statements read as "Theorem 2 is very powerful (at least partially) because the class of links it covers is large" and then it also has the main Theorem 1 as a simple corollary. The corresponding paragraph is rephrased as follows: Theorem 2 enables us to compute the braid index of any pseudo polyhedral link easily since the reduction number of such a link is easy to find. Furthermore, since the set of all double crossover polyhedral links with any given turn number is a subset of the set of all pseudo polyhedral links, the proof of Theorem 1 is a simple application of it, which we give below. In particular, for

specific double crossover polyhedral links constructed using either special template graphs such as regular graphs, or double crossover polyhedral links with specific turn numbers, their braid indices can be formulated using information only depending on the template graphs.

-Pg. 14. Can you provide more examples of knots and links in this class? Is there a database? See the response in 1 of Part 2 above.

-Pg 15. stylistic suggestion: replace the first sentence of Section 5 with "In this paper we provide explicit formula" to avoid repeating the same phrase. Done.

- Pg 16. Maybe replace: Thus we obtain ... with "the insights from the proof of Theorem 3...." Rephrased as: A proof similar to the proof of Theorem 3 leads to the following new result, which is summarized in Theorem 4.

- Pg 16. Define all notation used in Theorem 4. Done.
